# An integrated airborne transmission risk assessment model for respiratory viruses: short- and long-range contributions

**DOI:** 10.1098/rsif.2024.0740

**Published:** 2025-02-26

**Authors:** Andre Henriques, Wei Jia, Luis Aleixo, Nicolas Mounet, Luca Fontana, Alice Simniceanu, James Devine, Philip Elson, Gabriella Azzopardi, Markus Rognlien, Marco Andreini, Nicola Tarocco, Olivia Keiser, Yuguo Li, Julian W. Tang

**Affiliations:** ^1^ CERN (European Organization for Nuclear Research), Geneva, Switzerland; ^2^ Institute of Global Health, University of Geneva, Geneva, Switzerland; ^3^ Department of Mechanical Engineering, University of Hong Kong, Hong Kong SAR, China; ^4^ Strategic Health Operations, Operations Support and Logistic, Health Emergencies Programme, World Health Organization, Geneva, Switzerland; ^5^ Department of Civil and Mechanical Engineering, Università degli studi di Cassino e del Lazio Meridionale (UNICAS), Cassino, Italy; ^6^ Epidemic and Pandemic Preparedness, Health Emergencies Programme, World Health Organization, Geneva, Switzerland; ^7^ NTNU (Norwegian University of Science and Technology), Torgarden, Norway; ^8^ Respiratory Sciences, University of Leicester, Leicester, UK

**Keywords:** CERN Airborne Model for Indoor Risk Assessment, respiratory virus, COVID-19, airborne transmission, modelling, risk assessment

## Abstract

This study presents an advanced airborne transmission risk assessment model that integrates both short- and long-range routes in the spread of respiratory viruses, building upon the CERN Airborne Model for Indoor Risk Assessment (CAiMIRA) and aligned with the new World Health Organization (WHO) terminology. Thanks to a two-stage exhaled jet approach, the model accurately simulates short-range exposures, thereby improving infection risk predictions across diverse indoor settings. Key findings reveal that in patient wards, the short-range viral dose is 10-fold higher than the long-range component, highlighting the critical role of close proximity interactions. Implementation of FFP2 respirators resulted in a remarkable 13-fold reduction in viral dose, underscoring the effectiveness of personal protective equipment (PPE). Additionally, the model demonstrated that an 8 h exposure in a poorly ventilated office can equate to the risk of a 15 min face-to-face, mask-less interaction, emphasizing the importance of physical distancing and source control. We also found in high-risk or low-occupancy settings, that secondary transmission is driven more by overall epidemic trends than by the presence of individual superspreaders. Monte Carlo simulations across various scenarios, including classrooms and offices, validate the model’s robustness in optimizing infection prevention strategies. These findings support targeted interventions for short- and long-range exposure to reduce airborne transmission.

## 1. Introduction

The COVID-19 pandemic shed light on the importance of understanding the transmission mechanisms of respiratory pathogens. Out of many outcomes, one of the most controversial yet fundamental findings was the importance of the airborne mode of transmission. The inhalation of virus-laden particles has been found to dominate the infection chain and fuel the pandemic [1,2]. More recently, the World Health Organization (WHO) has updated their proposed terminology for pathogens that transmit through the air [3], dividing the process into (i) airborne transmission, occurring when infectious respiratory particles (IRPs) enter the respiratory tract through inhalation, at either short or long distances and (ii) direct deposition, occurring when IRPs follow a semi-ballistic trajectory at short-range, depositing on the facial mucosal surface.

Based on the new WHO terminology, the airborne route can be subdivided in two separate components [3]: (i) long-range airborne transmission which occurs via the inhalation of IRPs suspended in the room air (also referred to herein as background concentration); (ii) short-range airborne transmission which occurs via the inhalation of IRPs transported within the expiratory jet of the infected host, located within *close proximity* to the susceptible host during direct or partial face-to-face interactions. Throughout the pandemic, several analytical models such as the CERN Airborne Model for Indoor Risk Assessment (CAiMIRA; Henriques *et al*. [4]), were developed to try to predict the transmission risk in a given indoor setting and apply appropriate preventive and protection measures, yet focused exclusively on the long-range component. The short-range exposure had yet to be integrated into a combined short- and long-range continuum model, as proposed by Jia *et al*. [5]. Although identifying tailored mitigating measures for the long-range risk of infection is already helpful, the results may neglect the important contribution of an exposure from close proximity interactions with the infected host. Moreover, the homogeneous mixture assumption in long-range transmission models holds well when the susceptible host is far from the infector [6]. However, within *conversation distances*, the concentration is known to be higher due to the higher density of IRPs within the exhaled jet, contradicting the homogeneous mixture assumption.

In this paper, we propose to adopt the two-stage exhaled jet model to simulate the close proximity exposure of a susceptible host, in order to quantify the short-range inhalation dose, and combine it with the existing CAiMIRA model, into a multi-box (*short+long*) continuum, increasing the base of knowledge and respecting the new WHO terminology [3]. Providing such a complete and integrated tool will allow building engineers, facility managers, healthcare professionals and household individuals, to identify which measures or combination of measures are most suitable, thanks to a tailored risk assessment, ultimately leading to targeted investment.

## 2. Methods

The infection model presented in this paper is developed by extending an open source tool called the CERN Airborne Model for Indoor Risk Assessment (CAiMIRA) and focuses mainly on the integration of the short-range exposure into the existing long-range model published in Henriques *et al*. [4]. The source code and associated data for reproducibility is available in §5.

The methodology behind the infection model is split into five modules: emission (source), removal rate (dispersion or dilution), exposure (concentration over time), dose (inhalation) and risk (infection).

Many of the model variables (such as emission rate, removal rate and concentration) are considered for a given aerosol diameter D, as the dynamics in the room and the deposition efficiency in the respiratory tract, depend on the particle size. The resulting dose is then computed as the sum of both short- and long-range contributions, which is then followed by Monte Carlo integration over the corresponding particle size distribution (electronic supplementary material, equation S20). Furthermore, other parameters like viral load, interpersonal distance and infectious dose are considered as independent random variables, with statistical distributions (table 1) to account for their large variability and uncertainty; the related distributions are defined by the data available in the literature and are the main ingredients of a Monte Carlo sampling algorithm, allowing statistical estimation of transmission risk.

**Table 1.** Summary of random variables for the infection model used in [4] and added to cover the short-range exposure.

random (stochastic) variables					
variable	symbol	mean or [range]	s.d.	unit	fitting distribution model [ref]
breathing flow rate	${\mathrm{BR}}_{k}$				
seated	${\mathrm{BR}}_{\mathrm{se}}$	0.51	0.053	m^3^ h^−1^	log-normal [7]
standing	${\mathrm{BR}}_{\mathrm{st}}$	0.57	0.053		
light activity	${\mathrm{BR}}_{l}$	1.24	0.12		
moderate activity	${\mathrm{BR}}_{m}$	1.77	0.34		
heavy exercise	${\mathrm{BR}}_{h}$	3.28	0.72		
viral load	$vl_{\mathrm{in}}$	6.2	1.8	log_10_ RNA copies ml^−1^	Weibull kernel density estimation from dataset [8]^a^
mask efficiency	$\eta_{in,surgical}$	[0.25–0.80]	—	—	uniform [4]
	$\eta_{in,PPE}$	[0.83–0.91]			
	$\eta_{in,cloth}$	[0.05–0.40]			
IRP-to-RNA virus ratio	$f_{\inf}$	[0.01–0.60]	—	—	uniform [4]
infectious dose	${\mathrm{ID}}_{50}$	[10–100]	—	IRP^b^	uniform [4]
interpersonal distance	* x *	0.99	0.34	m	Gaussian kernel density estimation from dataset [9]^c^

^a^ Values truncated at $\log_{10}\left({\mathrm{vl}}_{\mathrm{in}}\right)=2$ and $\log_{10}\left({\mathrm{vl}}_{\mathrm{in}}\right)=10$

^b^ The dose can simply be expressed as infectious or viable virus

^c^ Values truncated at x=0.5 and x=2

The symbols used for the short-range model are identified with the subscript ‘SR’, and those related to long-range with the subscript ‘LR’. The model parameters are summarized in electronic supplementary material, table S1.

### 2.1. Multi-box model

In this study, we introduce a novel approach with a *multi-box* continuum model, where the susceptible host is either exposed to the exhaled jet within an interpersonal distance—short-range—or to the background concentration beyond this distance—long-range.

In addition to the interpersonal distance, the exposure time is another important factor to quantify the risk profile in a given setting. During an entire exposure scenario, the inhaled dose is the cumulative integral of the concentration profile over time, summing up the short- and long-range contributions.

#### 2.1.1. Short-range box

The short-range component of the model is derived from the exhaled jet concentration of IRPs in close proximity. The concentration profile is based on a two-stage exhaled jet model [5], which considers interpersonal distances, x, between 0.5 m (thus excluding intimate interactions) and 2 m [9,10], distance above which the jet is assumed to have diluted into the long-range (background) concentration. The 2 m limit is a model input parameter which can be changed, and serves as a mere indicator where short range stops and long range begins. Due to its large variability, the interpersonal distance x is considered as a random variable following the lognormal distribution of distances between two people standing in front of each other derived from Zhang *et al*. [9], truncated at a lower limit of 0.5 (cf. table 1).

The use of masks as source control is known to interrupt the forward momentum of the jet and project the air stream upwards, mainly due to the heat dissipated from the human body [11]. Hence, in this study the short-range component from an infected host wearing a face mask, is minimal at distance x and can therefore be neglected.

Here we assume the limit of integration of the diameter-dependent parameters up to $D_{max,SR}=$ 100 µm [12,13]—contrary to the long-range model [4] larger particle sizes are also considered because: (i) no evaporation factor is applied is short-range (do not have time to fully evaporate); and (ii) they stay within the jet before deposition (do not have time to fall down at short distances due to the jet dynamics).

In the following, we will first describe the ingredients of the short-range model in §2.1.1.1 to §2.1.1.3. The resulting concentration of IRPs at the location of the exposed individual will be given in §2.1.1.4, and its absorbed dose in §2.1.1.5.

##### 2.1.1.1. Jet profile and dilution

**Figure 1.**
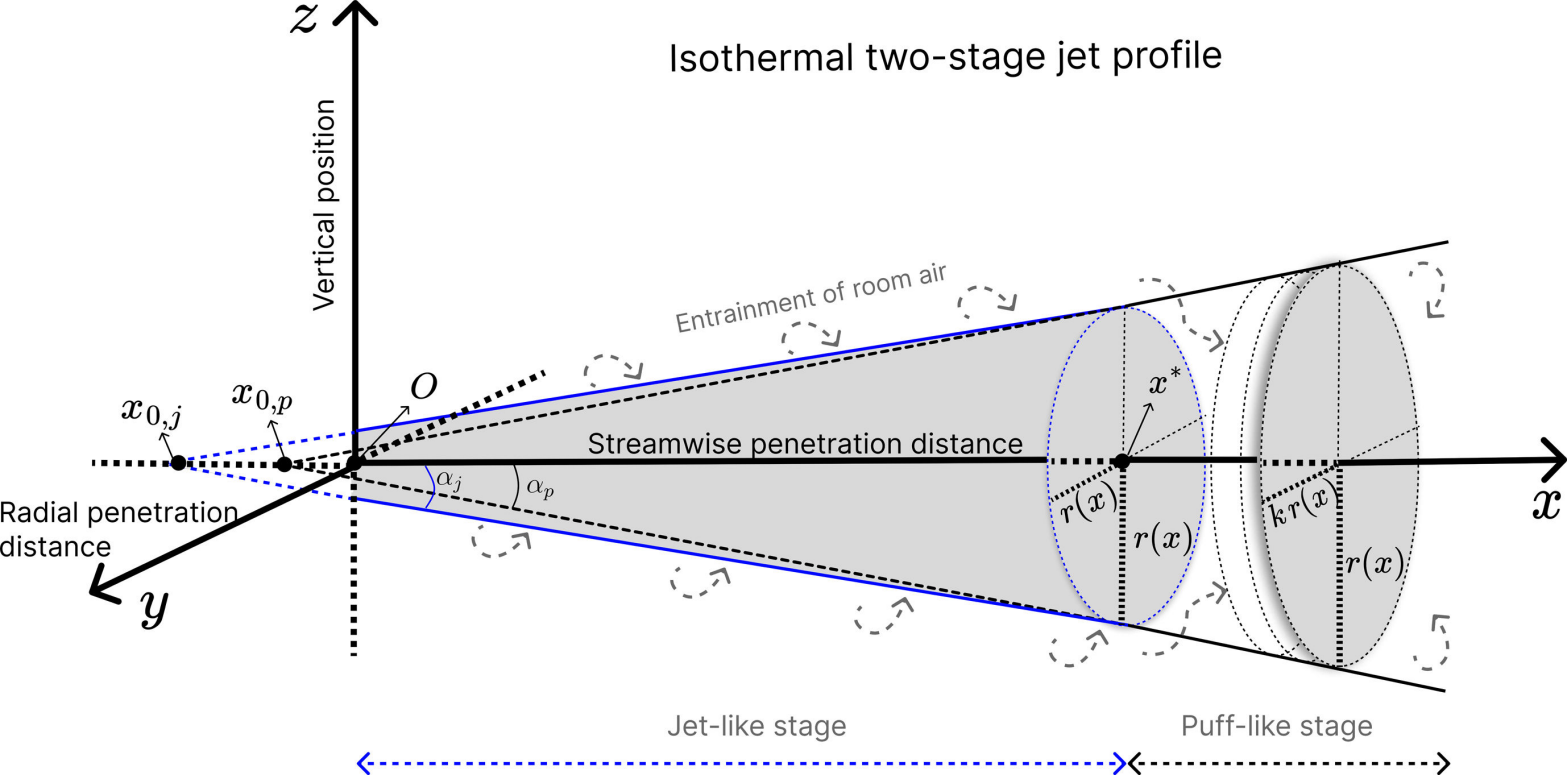
Schematic illustration of the isothermal two-stage jet.

The jet profile is determined on the basis of (i) a jet-like stage, where a steady-state expired jet is released from the source during expiration, forming a conic profile in the direction of the flow, and (ii) a puff-like stage, where the expiratory jet is interrupted to initiate the inhalation phase of the breathing cycle forming, which may be best described as an ellipse. The pattern repeats itself at each breathing cycle. The interrupted jet’s forward momentum produces a puff cloud, which is then pushed forward by the expiration flow of the following breathing cycle, producing *n* puff clouds one after the other (figure 1).

For simplification, buoyancy forces are neglected and an isothermal jet is assumed [5]. The eventuality of including thermal plume effects is discussed in §5.

The exhaled breath initiates its forward trajectory, increasing the jet’s volume due to continuous turbulent entrainment of ambient air as it develops, and losing its intensity (dilution) over the distance *x*. The dilution factor, S(x), corresponds to the ratio between the maximum concentration achieved at the jet origin and the concentration at distance x, derived exclusively from the increase in the jet’s volumetric flow rate. S(x) can range, theoretically, from 1 (x=0) to +∞ (x=∞).

The dilution factor at a given distance x is considered constant over time and can then be expressed as [5]


(2.1) $$\begin{array}{r} \begin{array}{r} S(x)=\left\{ \begin{array}{ll} \frac{2\beta_{r,j}(x+x_{0,j})}{D_{m}} & 0<x\leq x^{*},\\ S(x^{*}){\left[1+\frac{\beta_{r,p}(x-x^{*})}{\beta_{r,j}(x+x_{0,j})}\right]}^{3} & x\geq x^{*}, \end{array}\right. \end{array} \end{array}$$

where $x^{*}$ is the jet-puff transition distance; $\beta_{r}$ parameters are empirical and correspond to the radial penetration coefficients; $D_{m}$ is the average mouth diameter.

For simplification and in order to focus parametric studies on other variables, we assume a constant mouth opening with an equivalent hydraulic diameter $D_{m}=$ 20 mm and a 4 s breathing cycle, with a 1 : 1 inhale/exhale duration [5]. Additional details can be found in electronic supplementary material, SIII.1.

##### 2.1.1.2. Jet origin concentration

The initial emission concentration at the source (i.e. mouth/nose) per unit diameter, $C_{0,SR}(D)$ (in IRP ml m^−3^ µm^−1^), also known as the jet origin concentration, is estimated by considering the volumetric emissions of respiratory particles, multiplied by the viral load of the infected host (electronic supplementary material, SIII.2). For the emission of respiratory particles, we assumed the payload of genome copies is proportional to their volume (sphere) at saturated conditions, i.e. not desiccated. The jet origin concentration is assumed constant during the close proximity interaction and can be calculated using the following formula:


(2.2) $$\begin{array}{lcr} & C_{0,SR}(D{)}_{j}=E_{c,j}(D,f_{\mathrm{amp}})\cdot vl_{in}\cdot f_{\inf}, & \end{array}$$

where $E_{c,j}$ represents the volumetric particle emission concentration per unit diameter D (in $ml\;m^{-3}$), for a given expiratory activity j, as a function of the vocalization amplification factor $f_{\mathrm{amp}}$; $vl_{in}$ is the viral load inside the infected host’s respiratory tract (in RNA copies ml^−1^) and $f_{\inf}$ is the fraction of RNA copies that are viable to infection (i.e. the IRP to RNA ratio).

The time-dependent emission rate at short-range ($vR{,}_{SR}$, in IRP $\;h^{-1}$), is given by


(2.3) $$\begin{array}{lcr} & vR{,}_{SR}(D{)}_{j}=E_{c,j}(D,f_{\mathrm{amp}})\cdot vl_{in}\cdot f_{\inf}\cdot {\mathrm{BR}}_{k}, & \end{array}$$

where ${\mathrm{BR}}_{k}$ is the breathing rate for a given physical activity k, in $\;m^{3}\;h^{-1}$ .

In this study, we use data from the meta-analysis by Chen *et al.* [8] to define $vl_{in}$, fitted to a Weibull distribution truncated between $10^{2}$ and $10^{10}$ RNA copies ${ml}^{-1}$. The lower limit was determined based on the typical limit of detection (LOD) of reverse transcription polymerase chain reaction (RT-PCR) assays [14] whereas the upper limit is the maximum observed viral load value [8].

##### 2.1.1.3. Viability decay

Virus-laden particles, when exposed to environmental conditions, are found to lose their capacity to infect host cells before being re-inhaled by the susceptible host—this is called infectivity, or viability, decay [15].

The effect of the viability decay in the literature is mostly discussed for the Wells–Riley type long-range exposure, assuming an exponential decay with a half-life at the level of hours [16]. Due to the lack of data on virus deactivation in relation to droplet evaporation [17], the viability decay at short-range is generally neglected [10]. New evidence has emerged from Haddrell *et al*. [18], proposing a triphasic viral aerosol decay (TVAD) profile suggesting a possible rapid loss of viral infectivity at short distances, due to a quasi-instantaneous (less than 5 s) droplet efflorescence at low relative humidity (RH), ϕ, where SARS-CoV-2 IRPs were seen to lose about half of their infectivity in the first 30 s after exhalation. Hence, we propose the following linear viability decay factor at short-range ($\lambda_{SR}$) (electronic supplementary material, SIII.3):


(2.4) $$\begin{array}{r} \lambda_{SR}(x)=\left\{ \begin{array}{ll} 1-0.016\;t_{x} & 0<\phi \leq 40\%,\\ 1 & \phi >40\%, \end{array}\right. \end{array}$$

with $t_{x}=x/u_{0}$ (in seconds) the time at which the jet reaches a given distance x.

$\lambda_{SR}$ is also virus dependent [18], hence the values suggested in equation (2.4) may differ from pathogen to pathogen.

##### 2.1.1.4. Jet concentration

The concentration profile of the exhaled jet is computed assuming a continuum model connecting the boundary between the short- and long-range exposure [5], with the inclusion of a viability decay factor $\lambda_{SR}$. Due to the contribution of air entrainment from the surrounding environment, the background viral concentration in the room (i.e. long-range concentration) is a parameter of the jet concentration, which in turn is a function of time [4]. The short-range concentration is derived from the steady-state mass balance equation for IRPs in an exhaled jet and can be written [5,10]:


(2.5) $$C_{SR}(t,D)=C_{LR}(t,D)+\frac{1}{S(x)}\left(\lambda_{SR}(x)\;C_{0,SR}(D)-C_{LR}(t,D)\right).$$

During a given exposure time t, multiple short-range interactions can be defined in the model, and for each individual interaction, the expiration (related to the infector’s activity) may differ.

##### 2.1.1.5. Exposure and dose

The jet concentration integrated over the time duration of the short-range interaction determines the exposure of the susceptible host, $vD_{SR}$.

The combination of the exposure multiplied by the volumetric amount of air inhaled and the probability it stays within the respiratory tract, provides the dose absorbed over the exposure time given by


(2.6) $$\begin{array}{r} vD_{SR}(D)=\sum_{i=1}^{n_{SR}}\int_{t_{i}}^{t_{i+1}}\;C_{SR}(t,D)\;dt\cdot {BR}_{k}\cdot f_{dep}(D)\cdot (1-\eta_{in}), \end{array}$$

where $t_{i}$ and $t_{i+1}$ are the start and end times (in h) of each close proximity exposure, respectively; n is the total amount of independent exposures, in the same event; $f_{\mathrm{dep}}(D)$ is the (diameter-dependent) deposition fraction in the respiratory tract; and $\eta_{\mathrm{in}}$ is the inward mask efficiency (if any).

##### 2.1.2. Long-range box

The long-range component of the model is calculated using the current version of CAiMIRA [4]: single-zone, mass-balance model between the emission and removal of IRPs in the room air, leading to the analytical solution of the differential equation of the long-range concentration, integrated over the exposure time to obtain the dose of IRP (electronic supplementary material, SIV),


(2.7) $$\begin{array}{lrr} & vD_{\mathrm{LR}}(D)=\sum_{i=1}^{n_{\mathrm{LR}}}\int_{t_{i}}^{t_{i+1}}C_{LR}(t,D)\;dt\cdot {\mathrm{BR}}_{k}\cdot f_{\mathrm{dep}}(D)\cdot (1-\eta_{\mathrm{in}}), & \end{array}$$

where $C_{LR}$ is the long-range concentration (in IRP $\;m^{-3}$).

The concentration $C_{LR}$ results from the balance between the emission source(s) and removal mechanisms, depending on the occupants’ presence, as well as possible preventive and control measures. In this continuum model, the short-range viral emission can be seen as the source of the long-range virus concentration, with equation (2.3) providing the time-dependent emission of IRPs, with the addition of the reduction effect due to source control (e.g. the use of face covering) [19]. Since our previous study [4], new evidence emerged suggesting smaller particle sizes are responsible for long-range airborne transmission [20,21]. The limit of integration for the particle sizes at long range is approximately 20 µm ($D_{max,LR}$), which corresponds to a desiccated particle diameter up to approximately 6 µm , compared with the previous value of 30 µm (10 µm when desiccated).

As for the removal rate (in$\;h^{-1}$), the study considers four mechanisms [22]: ventilation (air exchange), particle removal (high-efficiency particulate air (HEPA) filtration—also known as equivalent ventilation), aerosol settlement (gravity) and viral inactivation (biological decay, UV radiation) (electronic supplementary material, SIV.1). Ventilation can be considered by dividing the fresh air flow rate by the volume of the room. In the case of natural ventilation, Bernoulli’s equation with the ideal gas law for single-sided buoyant flow is used, where the model takes the average outdoor temperature from historic meteorological data, and the user inputs the window opening dimensions and the average indoor temperature. The viability decay is computed based on the empirical formula from Dabisch *et al*. [23] with the ambient air temperature and relative humidity as input parameters. The removal via gravitational settlement is determined by applying the Stokes’ Law for aerosols [24].

### 2.2. Risk of secondary transmission

The risk of transmission depends on the total (absorbed) dose by the susceptible occupant(s), corresponding to the accumulation of both the short- and long-range exposures. These two components are added and have the same weight in the simulated event, i.e. the immunological effect of dose intensity (IRPs inhaled $\;s^{-1}$) is not considered. Equations (2.6) and (2.7) are then integrated over the respective diameter range


(2.8) $$\begin{array}{lcr} & vD^{total}=\int_{0}^{D_{max,LR}}vD_{LR}(D)\;dD+\int_{0}^{D_{max,SR}}vD_{SR}(D)\;dD. & \end{array}$$

To account for the aleatory uncertainties, some variables are treated as random. Table 1 summarizes the adopted distribution models and the related statistics. The resulting probability and cumulative density functions are obtained by plain Monte Carlo simulations (MCS) from those distributions.

The infection model only predicts transmission of secondary cases with the assumption that the incubation period is longer than the time scale of the simulation, which is between 3 and 4 days for COVID-19 [25].

The risk is calculated using a stochastic exponential dose–response model, without defining a minimum threshold intake dose to initiate infection, also known as single-hit or non-threshold models. Considering the lack of data for pathogens such as SARS-CoV-2, non-threshold models are generally used [6].

The dose–response for a non-threshold model, derived from [26] and adapted by [4] (electronic supplementary material, SV), is given by


(2.9) (2)P(I|vDtotal,ID50)=1−e−vDtotalID50ln⁡2⋅Tvoc⋅(11−HIexp),

where $P(I|vD^{total},{\mathrm{ID}}_{50})$ denotes the conditional probability of event I (infection) for given values of the absorbed and infection doses $vD^{total}$ and ${\mathrm{ID}}_{50}$, respectively; $T_{\mathrm{voc}}$ the reported increase of transmissibility of a variant of concern (VOC); ${\mathrm{HI}}_{\exp}$ is the host immunity of the exposed occupants. The model can simulate the effect of pharmaceutical interventions (e.g. vaccines) [4] by tuning the host immunity parameter with the vaccine effectiveness against a given virus, although for this study we focus on non-pharmaceutical interventions for the analysis (${\mathrm{HI}}_{\exp}=0$).

This model does not include a dispersion factor as possible superspreading effects can be represented by the variability of the viral load distribution [4].

#### 2.2.1. Probabilistic exposure of infected hosts

One can easily input the absolute number of infected occupants by assuming X people among the *n* occupants in the room are infected ($N_{\inf}$) and estimate the probability of infection based on that deterministic input. Our model also allows for an estimation of the probability of having at least one new secondary infection, assuming a probabilistic exposure.

The ‘infected/not-infected’ status of any single person participating in an event can be seen as a Bernoulli process of independent random variables, as a sequence of independent identically distributed Bernoulli trials. Hence, the number of positive cases among a population follows a binomial distribution [27], using as input the number of new cases and population (in a given region at a given time) and the ascertainment bias (AB) representing the level of unreported cases (electronic supplementary material, SVI). Since we discuss the probabilistic exposure retrospectively, we assume AB = 1.

By means of the total probability rule, the risk of having at least one new infection in an event with n occupants is given by [27]


(2.10) $$\begin{array}{lcr} & P(I\geq 1,n)=\sum_{i=1}^{n-1}P(I\geq 1|X=i)\cdot P(X,n), & \end{array}$$

where P(X,n) is the probability of a given individual being infected in a population of n occupants; P(I≥1|X=i) is the probability that at least one individual gets infected in the room with i infected hosts


(2.11) $$\begin{array}{lcr} & P(I\geq 1|X=i)=1-{\left[1-P(I|X=i,vD^{total},{\mathrm{ID}}_{50})\right]}^{n-i}, & \end{array}$$

where $P(I|X=i,vD^{total},{\mathrm{ID}}_{50})$ is the probability described in equation (3.2), with i<n.

## 3. Baseline scenarios

The model provides flexibility in terms of occupation profiles where both the infected or susceptible hosts can migrate in and out of the room at a given time, as well as the quantification of close proximity interactions, representing a possible real-life scenario. To be able to discuss the results in practical terms, we included a set of default activity profiles in terms of settings, vocalization and physical activities, occupancy, duration and ventilation.

The scenarios included in this study are as follows: naturally ventilated classroom with students seated in front of a teacher standing; shared office in a typical 8 h workday with 1 h lunch break; COVID patient treatment ward with one nurse for four patients providing care and treatment in 30 min rounds; and intensive care unit (ICU) housing patients with acute respiratory illness, each with a dedicated nurse providing care in an 8 h shift. In order to compare the impact of the interventions, by default the occupants are not wearing masks nor personal protective equipment (PPE). The infectors are all assumed to be infected with SARS-CoV-2 (Omicron). The detailed parameters of the scenarios are summarized in table 2.

**Table 2.** Baseline scenarios used to generate results. By default, no masks are used. The indoor temperature is assumed at 20°C. During the breaks, we assume the occupants leave the room and do not gather together in another indoor space, i.e. it considers a lapse of time where the occupants are not exposed to any other airborne IRPs. The short-range interactions are between the infected and one susceptible host. HCS (healthcare settings); HCW (healthcare workers); ACH (air changes per hour).

baseline scenario	activity	occupation	ventilation	volume (m^3^)	short-range interactions
shared office	office-type: — speaking 1/3 of the time, seated RH =60%	4 occupants; 1 infector: — 8 h workday exposure — 1 h lunch break	natural: 1 window opening of 1.6 × 0.2 m, summer season	50	speaking for 30 min in the morning with susceptible
classroom	training-type: — teacher: speaking, light activity — students: breathing, seated RH =40%	20 occupants (19 students); 1 infector (teacher): — 8 h class — 1 h lunch + 2 yard breaks	natural: 2 window openings of 1.6 × 0.2 m, winter season	160	speaking for 20 min in the morning; llistening for 15 min inthe p.m. with susceptible
patient ward	HCS-type: — patients: speaking 50%, sedentary — HCW: breathing, light activity RH =30%	5 occupants; 4 patients; 1 HCW: — 30 min rounds; — 8 h shift	mechanical: 3 ACH	48	15 min in close proximity each round
ICU	HCS-type: — patients: breathing, sedentary — HCW: breathing, light activity; RH =30%	12 occupants; 6 patients; 6 HCW: — 8 h shift	mechanical: 6 ACH	64	15 min in close proximity per hour: patient mainly breathing, speaking occasionally

## 4. Results

The results confirm that vocalization activities have a big impact on the emission rate, with speaking emitting two orders of magnitude more IRPs compared with tidal breathing, while louder vocalization activities (shouting/singing) yielded an increase of three orders of magnitude. The large variability is inherited from the viral load data (100 to 10 billion RNA copies ml^−1^), yielding large standard deviations in the results, which confirms the importance of shedding dynamics over the course of an infection [4]. Physical activity also has an impact on the emission rate, associated with the large variability of the viral load, although with less weight compared with vocalization activities.

**Figure 2.**
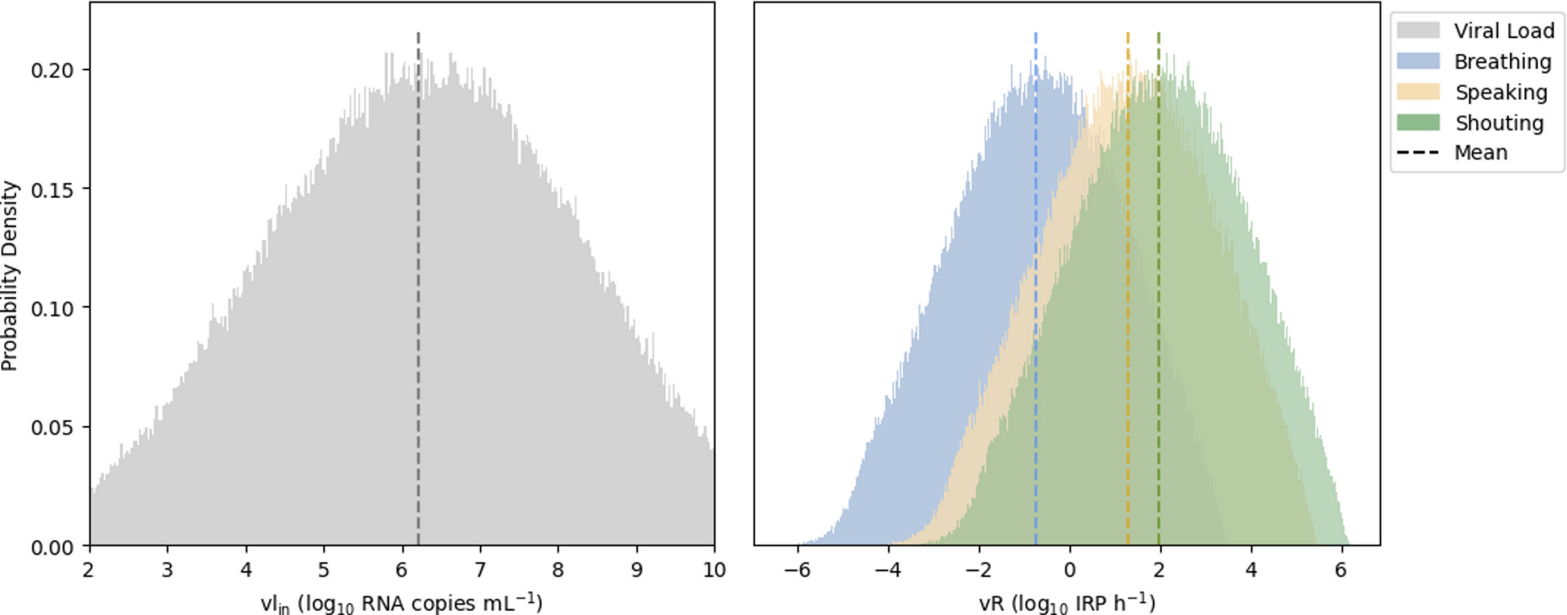
Emission rate distribution (right) for an infected host breathing, speaking and shouting, while undertaking sedentary physical activity (*standing*), compared with the genomic viral load distribution inside the host (left). Dashed lines correspond to the mean of the log_10_ values. The vertical axis represents the normalized frequency of the samples. The values are without the effect of face covering ($\eta_{\mathrm{out}}$ = 0). Plain MCS with 250 000 samples.

From a sedentary activity to heavy exercise, the emission increases from four to sixfold, respectively. The emission rate distributions are shown in figure 2 with a mean (s.d.) for breathing, speaking and shouting of −0.73 (1.84), 1.25 (1.84), 1.94 (1.84) log_10_ IRP h^−1^, respectively. With the update to the viral load distribution from Chen *et al.* [8], the viral emission rate $vR_{SR}$ fits now a Weibull distribution, compared with a custom kernal distribution in the previous version of the model [4] (electronic supplementary material, figure S1).

**Figure 3.**
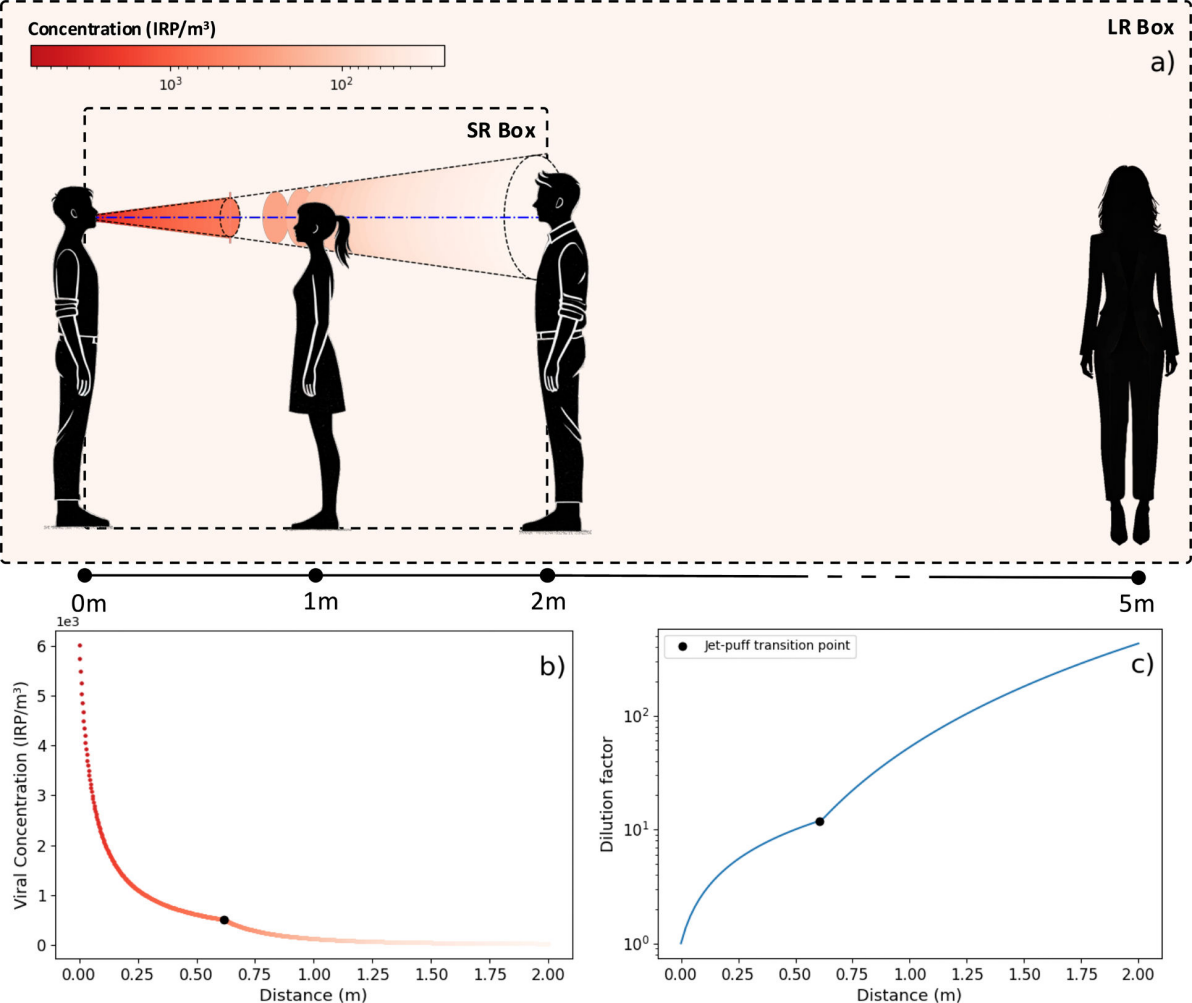
Illustration of the multi-box airborne transmission model and associated plots. The colour scale represents the change in viral concentration as a function of the distance from the infector. Both the infector and susceptible person are in light activity and speaking in a background (long-range) concentration of 10 IRP m${,}^{-3}$. The plot on the bottom right represents the jet dilution factor in this scenario. The black dots represent the point when the jet transitions into a puff.

Figure 3 illustrates the multi-box approach to this airborne transmission model. The concentration profiles and dilution effects can be visualized at scale with the isothermal two-stage jet diluting into the background (figure 3a), as well as the decrease in viral density (figure 3b) with the increase of the jet dilution (figure 3c). The jet origin concentration is proportional to the emission rate, with a mean concentration at the jet origin of 7 × 10^1^, 7 × 10^3^ and 3 × 10^4^ IRP m^−3^ for breathing, speaking and shouting, respectively.

**Figure 4.**
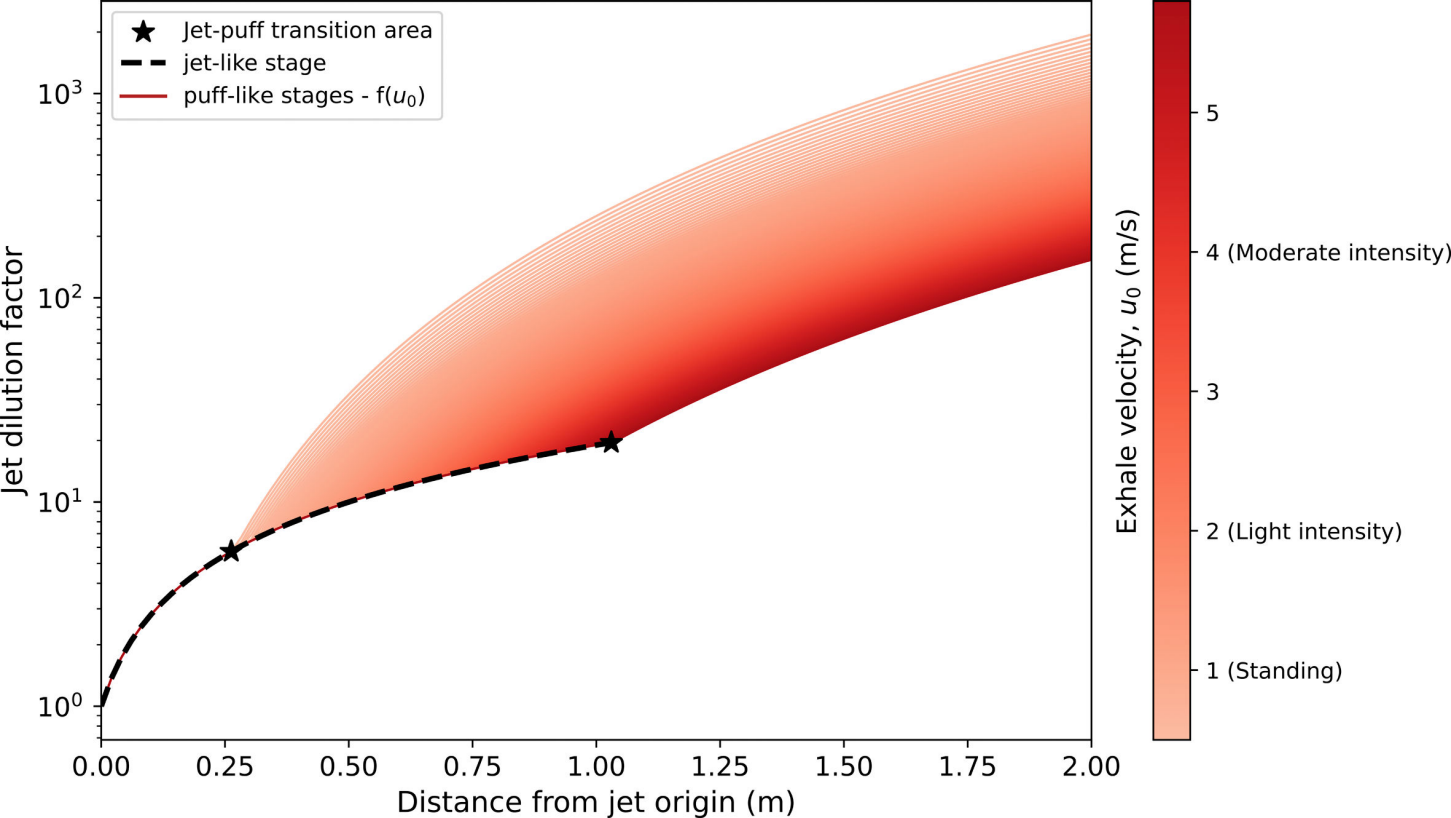
Dilution factor of the two-stage jet, as a function of the distance and exhale velocity. The black dashed line represent the jet-like stage which is independent of the initial exhalation velocity [5]. The red-based colour scheme reflects the puff-like stage of the jet ranging from different exhalation velocities, depending on the level of physical activity. The jet-to-puff transition point corresponds to the distance at which the transition from the black dashed line to the corresponding red-based colour lines occurs, for each exhale velocity. Mouth opening 20 mm and a 4 s breathing cycle with a 1 : 1 inhale/exhale duration is assumed.

At a distance of 1 m from the jet origin, the viral concentration reduces by two orders of magnitude for shouting and speaking (factor of 135 and 115, respectively), while for breathing resulted in a sixfold reduction, assuming the occupants are seated. At a distance of 2 m, the jet dilutes 100 to 1000 times, depending on the physical activity (figure 4).

The results of viral concentration and dose are shown in table 3. The patient ward scenario yields the highest cumulative dose inhaled by the healthcare workers (HCW) at the end of their shift (104 IRP). Running the same scenario but assuming the use of well-fitted FFP2 masks, the dose reduces to 8 IRP.

**Table 3.** Results of the peak concentration and inhaled dose for the different baseline scenarios (table 2), shown with mean and respective 90% confidence interval (displayed as μ [90% CI]). SR (short-range); LR (long-range). The mean peak concentration at SR corresponds to the value at a mean interpersonal distance of 1 m. These results are computed without the use of masks.

baseline scenario	peak concentration (IRP m^−3^)		inhaled dose (IRP)	
	SR	LR	SR	LR
shared office	90 [0–52]	2 [0–5]	20 [0–85]	6 [0–30]
classroom	289 [0–119]	3 [0–7]	67 [0–267]	8 [0–37]
patient ward	255 [0–147]	10 [0–21]	95 [0–408]	9 [0–43]
ICU	22 [0–13]	<0.1 [0– 2]	12 [0–48]	2 [0–10]

The classroom scenario reaches a peak jet concentration of 289 IRP m${,}^{-3}$, which is the highest of the four scenarios due to a longer vocalization activity during the short-range interaction (100% speaking versus 50% in the patient ward). The ICU scenario resulted in the lowest dose of them all (peak concentration of 22 IRP m${,}^{-3}$ and total dose of 14 IRP).

We studied the effect of the steady-state long-range concentration, normalized to the short-range concentration as a function of the distance to the infector. The results show that for vocalization activities the jet dilutes into background concentration (approx. 97%) after travelling 6.7 m, while for tidal breathing it is 4.6 m (electronic supplementary material, figure S2). Both cases yield a value greater than the 2 m threshold suggested in the methods.

The dose of infectious viruses absorbed by the susceptible person is the metric which determines the probability of secondary transmission within a given scenario. The inhaled dose is directly proportional to the integral of the concentration over the exposure time. In performing such risk analysis, the focus should not stop at the level of estimating the amount of virus in the air (concentration). It is equally important to consider the exposure time in order to determine the risk. The concentration at close proximity is two orders of magnitude higher than the steady-state background concentration, for the majority of the scenarios (table 3), although the relative time exposed at short range is normally lower than the time exposed to a long-range concentration.

In the office scenario, the dose contribution from short range ($vD_{SR}$) during a 30 and 15 min close proximity interaction, is 15 and 8 IRPs, respectively, whereas a dose ($vD_{LR}$) of 6 total IRPs is observed after spending 8 h exposed to long-range concentration alone.

**Figure 5.**
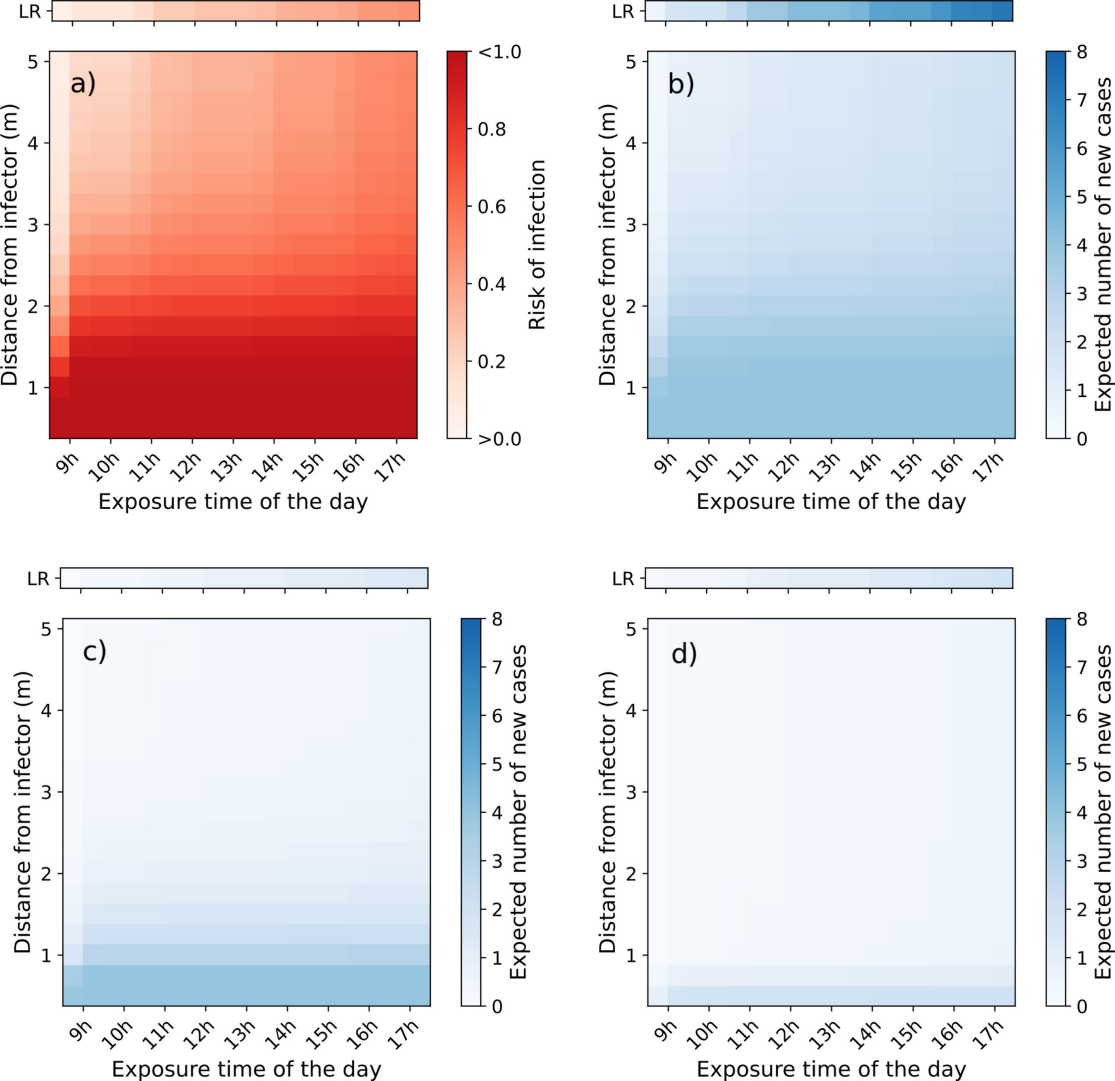
Heatmap of the cumulative risk of secondary transmission of a student in a classroom scenario: 8 h school day; 1 h lunch break and two yard breaks; one infector (teacher); four students exposed to short-range and 15 exposed to long-range; teacher: light activity; students: seated. Each of the four heatmaps is divided into two parts: the bottom part shows the results of the four students at short-range assuming a distance from 0 to 5 m, and the single row at the top represents those exposed to long-range (distance-independent). (a) Individual transmission risk without any intervention. (b) Expected number of new cases (attack rate) without any intervention. (c) Expected number of new cases when all the students wear FFP2 masks; (d) Expected number of new cases when the teacher (infector) is wearing a surgical mask as source control while the students are without any mask. The results in the last column on the right of each map corresponds to transmission risk/outcome of the scenario.

With this continuum model, we can evaluate what interventions have an effect at which range (short or long). By hypothetically following the exposure of the students in the classroom scenario, we show the cumulative risk profile as a function of the distance from the infected teacher over time (figure 5). For this analysis, we assume 4 out of the 19 students are in close proximity with the infected teacher (short range) and the remaining 15 exposed to the background concentration (long range).

The individual risk at short range increases as the distance decreases, independently on the exposure time, ranging from approximately 100% at distances below 1 m and 70% at 2 m (figure 5a). At long range, the risk becomes considerable after a couple of hours of exposure: 35% risk after 4 h (figure 5a). By contrast, the absolute number of expected new cases at long range is higher (7 out of 15), compared with short range (3.2 out of 4 at 2 m) (figure 5b). By adding PPE, such as FFP2, the impact is felt at long range, reducing the cases by a factor of 5 (from 7 to 1.4), although the transmission risk still remains high at very close proximity with three new cases at 1 m (figure 5c). If the infected host would use a surgical-type mask as source control, the risk of transmission to the students would decrease by a factor of 6 at short range (from 4 to 0.65 at 1 m) and a factor of 4 at long range (from 7 to 1.9) (figure 5d). Source control measures are rather more effective in reducing the risk at short range.

All the results presented above assume a deterministic number of infected occupants. By applying instead a probabilistic exposure in terms of infected occupants (equation (3.3)), the results differ. For the classroom scenario, with an occupancy of 20 people, the risk of secondary transmission assuming (deterministically) one infector is 13%. With the probabilistic assumption during an incidence rate of 100 new cases per 100 000 inhabitants, and assuming the exact same exposure scenarios, the risk increases to 23%. For a lower incidence rate (10 per 100 000) the risk decreases to 2.5%. This confirms the importance of whether or not the occupants are infected, in fuelling the chain of transmission. For scenarios with lower occupancy, this effect has more weight. Taking the shared office scenario, with one deterministic infector the risk is 11.5% and with the probabilistic assumption (incidence rate of 100 per 100 000) it reduces to 1.7%.

## 5. Discussion

This study aims to simplify the computation of a complex process by introducing a new analytical approach for determining both short- and long-range components of the new WHO terminology [3]. This work improves the accuracy of the existing model, which is used in tools such as the WHO’s ARIA app [28].

While more detailed input parameters could be considered, we maintained certain assumptions to balance computation efficiency with relevance to the objective. For example, ambient movements, such as occupants walking or doors opening, can affect virus spread. However, incorporating such dynamic effects would require computational fluid dynamics, which conflicts with the goal of creating a simple, user-friendly tool with conservative simplifications.

The long-range part of the model was benchmarked using the results of epidemiological investigations, and the results were published in a previous study [4]. Focus is given to the newly introduced short-range box, which results in a higher model accuracy in close proximity to the infected host.

The viability decay at short-range ($\lambda_{SR}$) shows to have minimal effect within typical conversational distances. Despite a steep slope in low humidities (equation (2.6)), the time needed for the virus to be inhaled remains too small for this effect to have any significance. Even for a sedentary activity (e.g. seated or standing), the exhale velocity of 0.5 to $1\;m\;s^{-1}$ is still too high. The viability decay at short ranges can therefore be neglected in this model.

In similar efforts to model viral emissions, Jones *et al*. [29] predicted a mean emission rate of $10^{-1}$ viable virus h^−1^ for a 75–25% breathing-speaking activity, compared with 5  IRP $\;h^{-1}$ from this study for the same expiratory activity. For 100% breathing, our model yields much lower values (0.19  IRP $\;h^{-1}$), showing the importance of vocalization in the emission source. The slight disparities in literature may have several sources, e.g. host immunity, air sampling techniques and plaque forming unit (PFU) assay procedures, although it is most likely linked to the tuning of the IRP-to-RNA ratio ($f_{inf}$) parameter. Data from the COVID-19 human challenge clinical studies [30] derive an emission rate ranging from $10^{0}$ to $10^{4}$ IRP h^−1^ [29–31], which falls within the span of results observed in our study.

Epidemiological studies on short-range exposure are limited, probably due to challenges in separating viral transmission modes (airborne, direct deposition, fomite) and the lack of human behaviour data [32]. With the updated terminology and clear definitions for short-range transmission, this may improve in the future. Despite the absence of *in vivo* data to fully validate the transmission risk, the model’s mechanistic behaviour remains robust in comparison with the literature.

The assumption that the number of virions is proportional to particle volume has been adopted by other authors [5,10,33]. However, this approach overlooks the possibility of individual IRPs containing multiple infectious viruses (*multi-virion* particles) [34], which would increase dose intensity and reduce the immune system’s neutralizing effect, impacting the transmission risk. Additionally, recent evidence indicates a non-proportional relationship, observing a higher virion density in smaller particles [21]. Therefore, this proportionality assumption is left as a limitation that requires refinement as new evidence emerges.

The vocalization activity seems to have a much higher influence on the emission rate, compared with the impact of different physical activities. From breathing to speaking, the emissions increase two orders of magnitude, which would indicate an infected host in a noisy bar is likely to emit more virus than someone exercising at the gym.

The results of the jet dilution factor and streamline penetration distance match those from [5] (figure 4). During the jet stage ($x<x^{*}$), the dilution factor is independent on the exhalation velocity for a given breathing cycle. The dilution effect increases significantly once the jet is interrupted by the end of the cycle, passing from a $1/x^{2}$ to a $1/x^{4}$ power law (electronic supplementary material, equation S9). The results show the lower the physical activity, the quicker the jet will dilute into the background concentration (figure 4, electronic supplementary material, figure S2). In other words, having a low breathing rate (and consequently low exhale velocity) will anticipate the formation of the puffs which, in turn, increases the dilution effect at the same distance, compared with other activities. Failing to model the puff dynamics, and relying solely on conic jets, would result in large underestimation of the dilution factor, namely at low to mid physical activities (figure 4).

**Figure 6.**
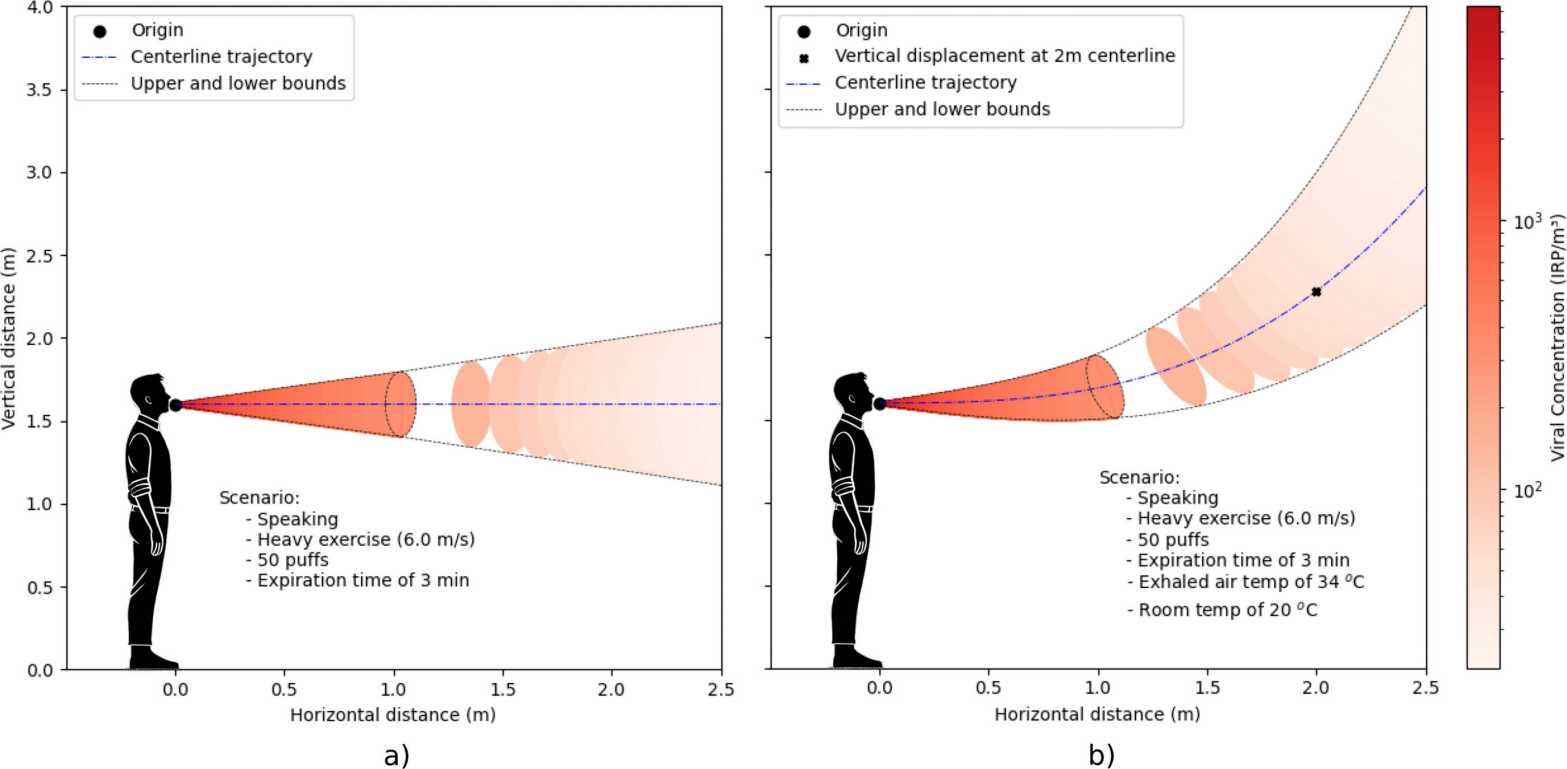
Centreline profile of the two-stage expiratory mouth jet, in the case of heavy exercise, with (a) isothermal assumption and (b) the effect of buoyancy. Each ellipse corresponds to a single puff due to the interruption of the jet phase. Initial position of the jet origin is 1.6 m; in (b), the vertical distance of the centreline at 2 m from the jet origin is 2.1 m. Jet temperature of 34°C and ambient temperature of 24°C.

The model assumes an isothermal jet. Including the effect of buoyancy would, in reality, divert the jet upwards and possibly reduce the concentration at the breathing zone. A simplified analytical approach can be used to estimate this effect, based on the Archimedes number introduced by Hagström *et al.* [35], for a jet exhaled into a well-mixed environment with no stratification (dT/dy=0),


(6.1) $$\begin{array}{lcr} & \frac{z(x)}{\sqrt{A_{0}}}=\psi \frac{K_{2}}{{K_{1}}^{2}}Ar_{0}{\left(\frac{x+x_{0}}{\sqrt{A_{0}}}\right)}^{3}, & \end{array}$$

where z(x) is the vertical (upward) travel of the jet, over the distance x (in m); $K_{1}$, $K_{2}$ and ψ are jet characteristic coefficients; $Ar_{0}$ is the Archimedes number at jet origin and $A_{0}$ is the mouth opening area (electronic supplementary material, SIII.1). This thermal approximation was not benchmarked with literature, although it is used here to discuss the boundaries between short- and long-range transmission. For an activity ranging from heavy exercise ($u_{0}=$ 6 m s^−1^ ) to standing ($u_{0}=$ 1 m s^−1^) the thermal effect of the jet yields a 0.5 to 16 m vertical lift of the centreline trajectory at a distance of 2 m from its origin (figure 6).

Higher initial velocity of exhaled air reduces vertical displacement, hence heavy exercise would result in the smallest deflection (figure 6). For face-to-face conversations, this effect reduces the risk [11]; however, in other scenarios like supermarkets where cashiers are positioned lower, the thermal plume would raises the jet towards the customer’s breathing zone. Adding thermal effects would increase the level of complexity and a numerical approach would be required to solve it; nonetheless, the isothermal approximation still provides a good estimate, especially in displacement-ventilated rooms [36], although for thermally stratified environments the approximation is less accurate [36].

The model does not consider using the nose as the expiration source. Breathing from the nose would deviate the initial velocity vector downwards, towards the chest and away from the susceptible host, by 45° [36]. Adding angles to the jet profile would induce complexity to the model, hence it is not included in this study.

For a steady-state isothermal jet, a Gaussian distribution may be used to describe the radial concentration profile [37]. For interrupted two-stage jet models, the same assumption has yet to be studied. Although, using a constant (flat) radial distribution in this study, preserving the same mass and momentum fluxes, is a conservative simplification [5].

According to Liu *et al*. [38], the infection risk at short range is sensitive to the interpersonal distance, the human posture and the indoor air stratification. The authors draw to the attention that the 2 m social distance rule may be valid only when neglecting thermal stratification [38], indicating a higher risk beyond this distance. In our model, the effect of stratification is indirectly covered in the long-range component of our continuum model (equation (2.5)), where for poorly ventilated areas allowing for stratification, the risk will be significantly higher, even at distances greater than 2 m. The majority of the interpersonal face-to-face interactions are found to be within 0.5 and 2 m [9], therefore, even if there is a non-negligible jet concentration beyond 2 m, the chances that two occupants interact face-to-face is statistically low. Nonetheless, this model is flexible enough to assume distances beyond 2 m (figure 5). The physical activity has an impact on the jet concentration for distances up to 0.5m; beyond that it is dominated by the expiratory activity alone (electronic supplementary material, figure S2).

Out of the four scenarios (table 3), the COVID patient ward unsurprisingly reflects the worst case, mainly due to the fact that one can deterministically assume the presence of infected people emitting virus and the necessity for the HCW to interact with them in close proximity. The long-range component of the dose in the same scenario is 10 times lower than the short-range component, due to the presence of mechanical ventilation. This could indicate the likelihood of transmission to HCW is governed by the close proximity interactions, yet the long-range component is still substantial—at the level of the infectious dose used in the human challenge study (10 IRP) [30]. Optimization scenarios might be necessary to reduce the risk, e.g. reducing the close interactions to a strict minimum, providing PPE like respirators or other measures like surgical masks, which work both in short and long range and provide inward and outward protection [19]. Surgical masks have been shown to reduce outgoing emissions by two to fourfold or 25–90% [19,39], depending on the particle size bin.

Using our model, the use of FFP2 respirators in the patient ward scenario induces a 13-fold reduction in the viral dose inhaled by the HCW (104 to 8 IRP). The ICU scenario, on the other hand, is quite resilient since the patients are mostly sedated and incubated on closed-loop ventilators, thus emitting less viral load to the air, coupled with good ventilation. In the shared office, the risk spent in a poorly ventilated space during an 8 h workday without any close proximity interactions is comparable to a 15 min face-to-face conversation with the infected host, demonstrating once more the importance of physical distance and/or face covering as source control in infection prevention and control.

When it comes to viral transmission, a lot depends on the specific and cross-reactive immunological history of those exposed, especially nowadays for COVID-19, when most people have been exposed to different SARS-CoV-2 variants, through natural infection or vaccines. Various studies indicate that the combination of past infection plus vaccine course plus booster with a different vaccine (so-called ’heterologous booster’), provides a robust and wide-ranging protection against severe disease, death and even symptomatic infection [40]. This means the infection risk is highly host-dependent, with different parameters to model, making certain approximations/simplifications like the idea of one single *quantum of infection* from Wells–Riley seems unrealistic as a predictive tool. Our model, however, can include the effect of possible immunological responses which skews the risk for different hosts. To include host sensitivity in the model, we fine-tuned the infectious dose (${\mathrm{ID}}_{50}$) by the effect of a host-immune response following vaccination, impacted by the different variants of concern. Host immunological aspects are clearly more complex than a single vaccine efficiency value in the model, which is considered a limitation of our study. Other host immunology scenarios are in the pipeline for future model improvements, such as: (i) exposed to a first-time natural *primary* infection with no pre-existing immunity, resulting in lower infectious doses and becoming infectious before symptom onset until viral clearance by IgG/IgA antibodies; or (ii) natural re-infection or vaccinated exposed hosts, which would require a high infectious dose and reduce the viral emission. This in turn will also have an effect when using such event-specific transmission models to population-based epidemiological models where secondary infection chains within the same exposure group depend highly on the host immunology scenario. Moreover, we assume an infection could be initiated (albeit with a low probability) as soon as a single IRP is absorbed in the respiratory tract and binds to the receptor. Alternative approaches are possible, such as multi-hit models where a minimum threshold dose is required before the risk starts to increase above zero. Different models should include incubation periods, shedding dynamics and clinical severity, which may differ from host-to-host, impacting not only the dose–response but also the viral load emission rates, which are not included for the time being and would be considered in a future update.

The results in this study also highlight the importance of rigorously following the hierarchy of controls for infection, prevention and control [41]. Eliminating or reducing the hazard at the source (i.e. source control measures) are more effective then prescribing PPE and should have priority (figure 5). Jumping straight to the conclusion of prescribing respirators before any source control or engineering control measure is sometimes not the most efficient way of breaking the chain of transmission. Nonetheless, it is important to mention that respirators are effective PPE devices, designed to protect the wearer [42,43], therefore effective at an individual level only. For settings with relatively high occupancy (e.g. a classroom) the overall attack rate is reduced by focusing on interventions to limit the viral content in the room (figure 5a,b), as more occupants are exposed to background concentration for longer periods, compared with those experiencing high short-range concentrations but for shorter intervals. Source control measures are effective in reducing the risk at both short and long range, whereas PPE is more effective at long range (figure 5c,d).

For the shared office scenario, one single super-emitter with $10^{9}$ RNA copies (approx. 90th percentile [8]) leads to a 90% transmission risk. Using a probabilistic approach, similar risk levels are reached only with high incidence rates (4000 per 100 000 inhabitants), comparable to the peak of COVID-19 infections in Geneva [44]. This indicates that when applying probabilistic exposure (incidence rate) to the model, the transmission risk in low-occupancy settings seems to be driven more by the likelihood of having at least one infector in the room than by their viral shedding capabilities. For high risk indoor settings (e.g. crowded, poorly ventilation events with no source control measures) the first term of equation (3.3) will tend to one. Thus, the total probability (P(I≥1,n)) is equal to the second term (P(x,n)), which depends exclusively on the epidemiological characteristics leaving again the secondary transmission risk driven by the presence of infected attendees, irrespective of their viral shedding. We conclude that in high-risk or low-occupancy settings, secondary transmission is driven more by overall epidemic trends than by the presence of individual superspreaders, indicating that pathogen prevalence can serve as a risk indicator, supporting the use of cut-off values as guideline thresholds, e.g. for massive indoor gatherings. This outcome is compatible with recent findings where only 3% of index cases were superspreaders [45].

## Data Availability

The source code for the CAiMIRA model and data to reproduce the results are available under Apache 2.0 open source license from our code repository. The code is also available in Zenodo [46]. The figures were generated using Matplotlib [47]. Supplementary material is available online [48].
